# Complete mitochondrial genome and the phylogenetic position of the giant oarfish (*Regalecus glesne*)

**DOI:** 10.1080/23802359.2019.1623124

**Published:** 2019-07-10

**Authors:** Yue Yu, Xin Peng, Can-Min Yang, Xiao Chen, Shaobo Chen, Song Qin

**Affiliations:** a College of Marine Sciences, Shanghai Ocean University, Shangahi, PR China;; b Zhejiang Mariculture Research Institute, Wenzhou, PR China;; c College of Marine Sciences, South China Agricultural University, Guangzhou, PR China

**Keywords:** *Regalecus glesne*, Lampriformes, mitogenome

## Abstract

The mitochondrial genome of *Regalecus glesne* was firstly elucidated and analyzed in this study. It had a double-stranded DNA molecule with the length of 16,536 bp and was made up 37 genes (13 protein-coding genes, 2 rRNA genes, and 22 tRNA genes) and one control region. Furthermore, the phylogenetic result demonstrated that *R. glesne* clustered with *Trachipterus trachypterus* and *Zu cristatus*. The complete mitochondrial genome provided in this work would be helpful for the genetic elucidation of the evolution of Lampriformes and other orders.

*Regalecus glesne* (Regalecidae, Lampriformes) is the longest bony fish in the world (Foot 2000). It is globally distributed in the pelagic zone of the marine, which is a oceanodromous species following its main food source. Notwithstanding distributed widely between 72°N and 52°S, the most suitable range is the tropics to middle latitudes (Schmitter-Soto 2008). Giant oarfish is not fishing for market, though being a bycatch occasionally, and as such it has been sold (Burton and Burton 2002).

Herein, we firstly report and analyzed the complete mitochondrial DNA genome of *R. glesne*. The specimen of *R. glesne* (Voucher: SY2015102354) was collected on the fish pier from Sanya, Hainan Province. The experimental protocol of DNA extraction and data processing methods of raw data was according to the method described by Li et al. (2015). The complete mitogenome had a double-stranded DNA molecule with the length of 16,536 bp (GenBank accession no.MK209627), containing 37 genes (13 protein-coding genes, 2 rRNA genes, 22 tRNA genes) and one control region with a typical mitogenomic organization and gene orders in line with other vertebrates. The overall base composition of the *R. glesne* mitogenome in descending succession was: 28.5% T, 28.1% C, 25.3% A, and 18.1% G, with a slight A + T bias of 53.8%.

Phylogenetic reconstruction based on the complete mitochondrial genome shared by other order species was inferred from the maximum likelihood (ML) method and ML bootstrap analysis using MEGA5; bootstrap probability values were calculated from 1000 replicates. Excluding *R. glesne*, 15 species of supplementary orders were selected to construct the phylogenetic tree with 12 protein-coding genes encoded on the H-strand (except for *ND6*). Additionally, *Carapus bermudensis* and *Sirembo imberbis* reckoned as the out-group.

The phylogenetic result divided the 16 fishes into five groups and all orders were monophyletic. Phylogenetic analysis showed that *R. glesne* clustered to *Trachipterus trachypterus* and *Zu cristatus*, which indicated the phylogenesis classification of *R. glesne* was consistent with the morphological result (Figure 1).

**Figure 1.**
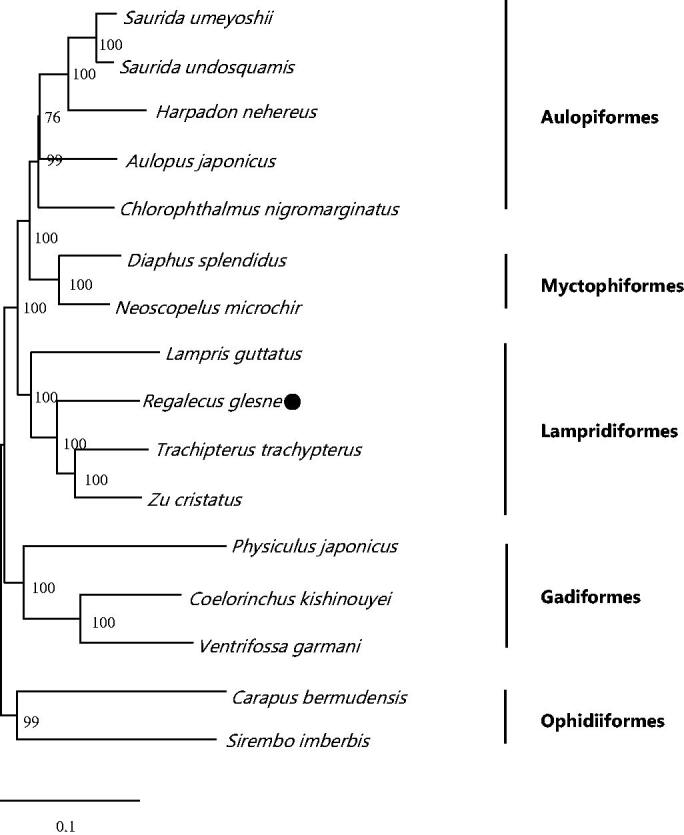
Phylogenetic position of *Regalecus glesne*. *Carapus bermudensis* (NC_004373) and *Sirembo imberbis* (NC_008123) were selected as the outgroup. ‘Black dot’ is *R. glesne.* The other species were *Ventrifossa garmani* (NC_008225), *Coelorinchus kishinouyei* (NC_003169), *Physiculus japonicus* (NC_004377), *Zu cristatus* (NC_003167), *Trachipterus trachypterus* (NC_003166), *Lampris guttatus* (NC_003165), *Neoscopelus microchir* (NC_003180), *Diaphus splendidus* (AP002923), *Chlorophthalmus nigromarginatus* (NC_027654), *Aulopus japonicus* (NC_002674), *Harpadon nehereus* (JX534239), *Saurida undosquamis* (KJ511779) and *Saurida umeyoshii* (NC_024836).
